# Lessons in automation of meat processing

**DOI:** 10.1093/af/vfac022

**Published:** 2022-04-30

**Authors:** Mark Seaton

**Affiliations:** Research & Development Manager, Scott Technology Ltd, Dunedin, New Zealand

**Keywords:** automation, meat processing, robotics

**Implications**

• The challenges in developing and adopting automation for meat processing are high but can be overcome.

• Great care is needed in the justification of automation. The benefits are not always obvious.

• Practical implications at the processing site need consideration, such as machinery size and maintenance skills.

• There are many automation development considerations that are unique to meat processing.

## Introduction

**Figure 1.**
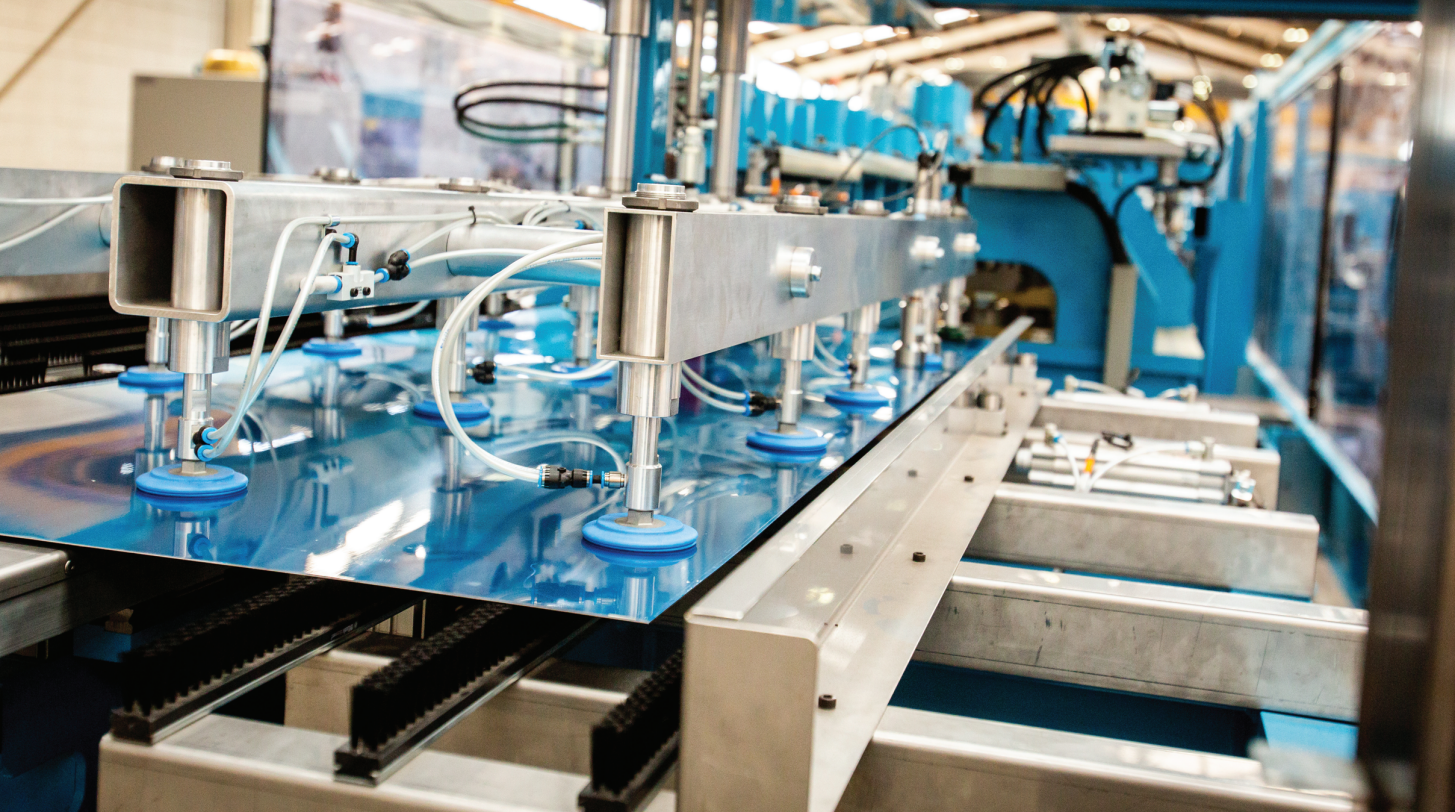
A photograph of a Whiteware appliance manufacturing system, which was Scott Technology’s primary focus until the U.S. recession in 2001.

Scott Technology Ltd (Scott) is a New Zealand-based company that specializes in developing and manufacturing automated production systems. With a history spanning 108 years, the company has shifted its focus many times. One of the most substantial focus shifts was in 2001 when the U.S. whiteware appliance industry (Figure 1) that Scott relied on suffered a major downturn and Scott set upon a diversification strategy.

In the most significant outcome of diversification, Scott formed a joint venture with New Zealand processor Silver Fern Farms Ltd (at the time known as PPCS Ltd). This joint venture (JV)—Robotic Technologies Ltd—had the ambitious plan of automating the entire lamb boning room. It was envisaged by Scott that this would take a year to design, build, install, and have running perfectly in production… Silver Fern Farms was (fortunately) able to comprehend the enormity of this task, and a lesser target was set.

**Figure 2.**
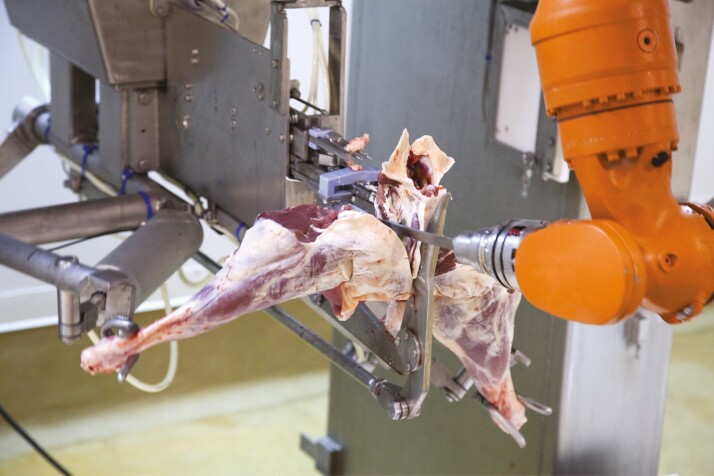
Lamb Hindquarter Boning was Scott’s first challenge in meat processing.

The first task set was to automatically (robotically) bone out the lamb hindquarter—separating the two legs from the aitchbone (Figure 2). It was an incredibly challenging task that stretched the capabilities of the technologies of the day and nothing short of amazing that the team involved was able to achieve it. But then things took an interesting turn with employees in the plant.

With four Hindquarter machines in operation in Silver Fern Farms’ Silverstream boning room, the workers alongside them performing the same task took up the challenge to improve their own performance. Within weeks, their yield had improved to a level that was not attainable in the machines, and with no obvious way to improve, the technology was taken out of production.

## Justification

As highlighted in the example above, automating a task is only one part of the challenge. Great care is needed in the justification of automation, but getting it right and the benefits can be very compelling. This can be a complex area to understand, and so I will share some of my insights from working both in development and in sales at Scott Technology.

### Labor

**Figure 3.**
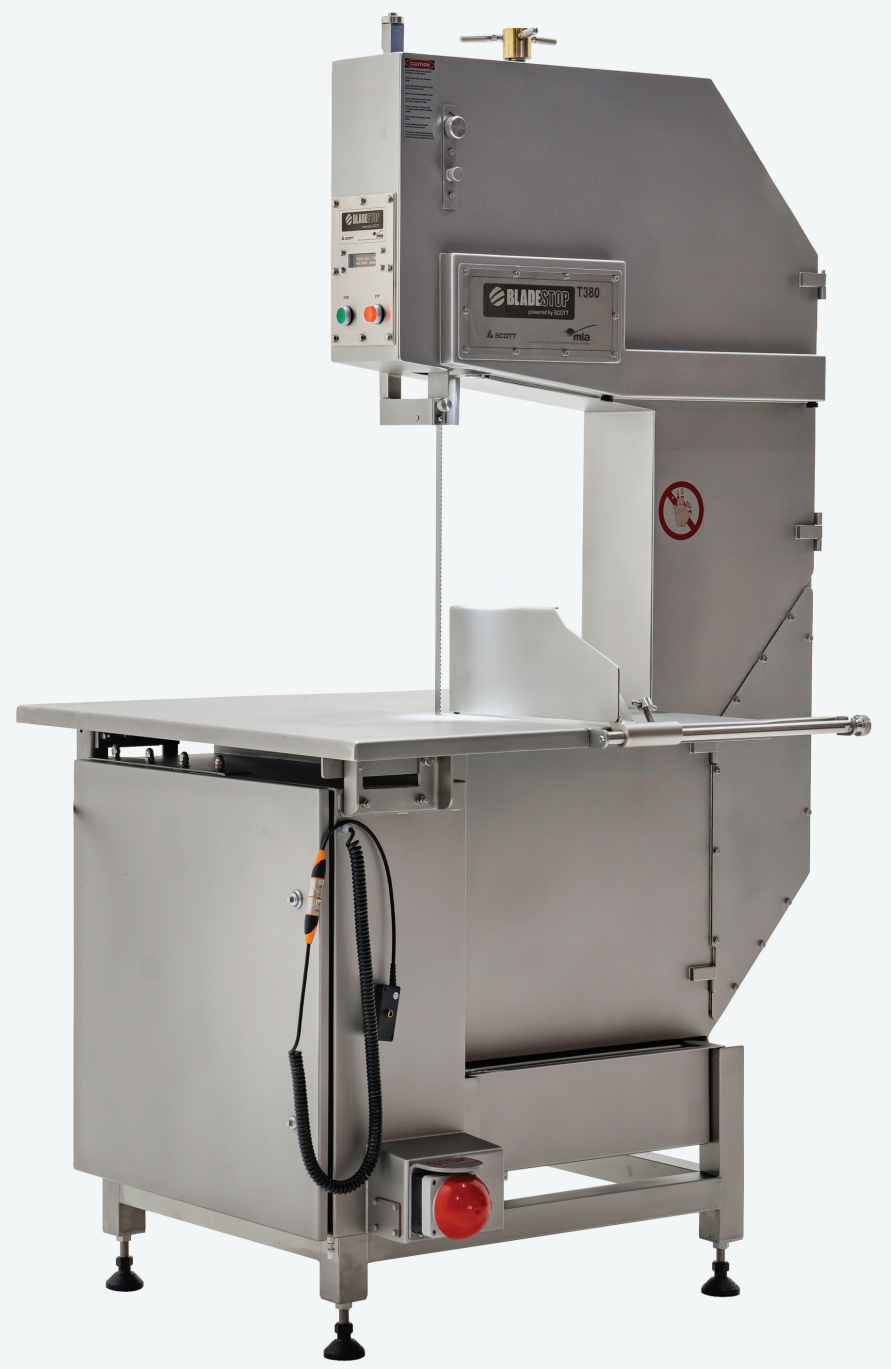
The Bladestop bandsaw that stops within milliseconds to minimize injury to operators.

**Figure 4.**
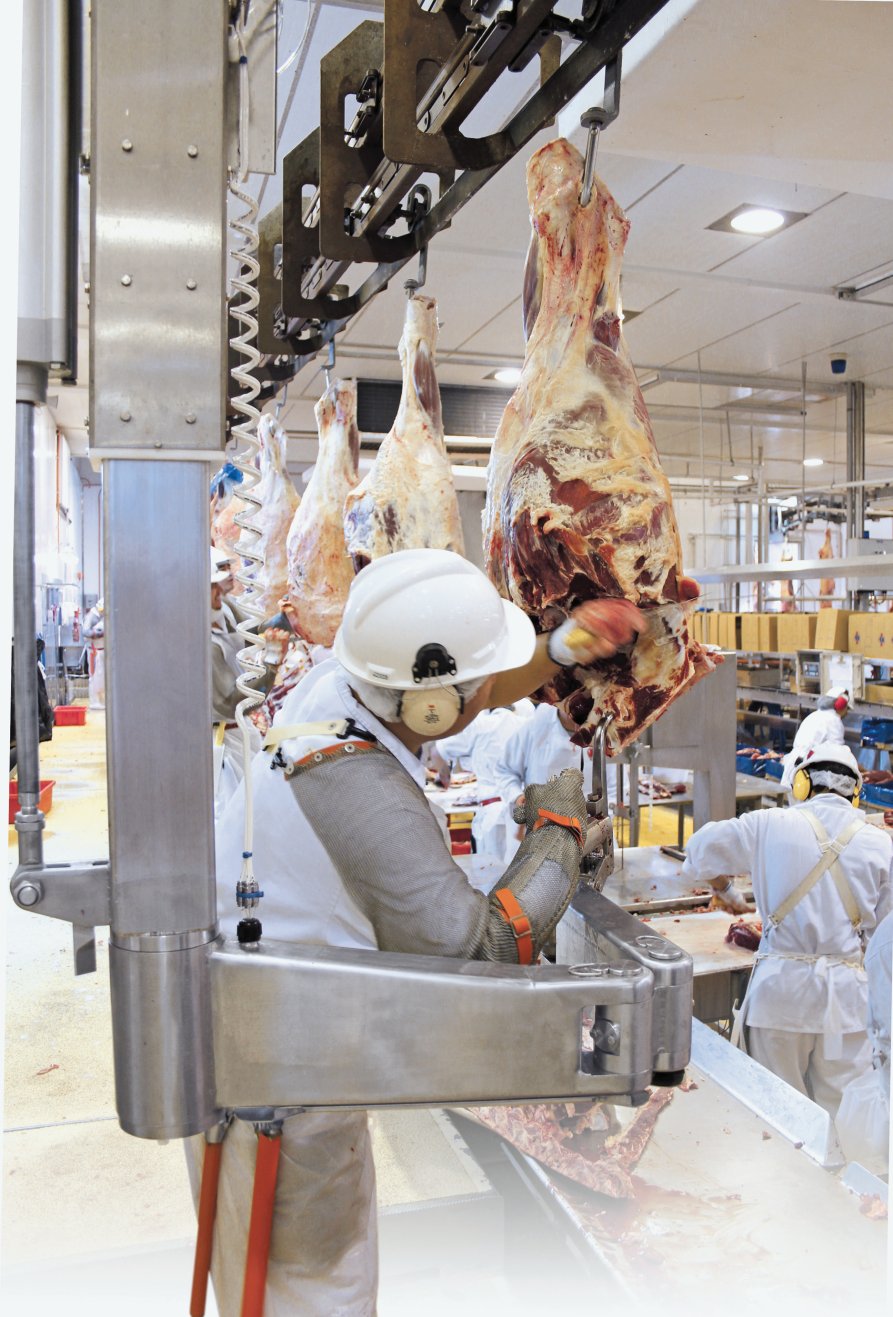
The Beef Boning Unit eliminates the heavy pulling when boning out Beef aitchbones and knuckles.

When most people think of automating a task, it is generally to replace a person. But rarely does labor replacement alone justify the cost of purchase. As a rule of thumb, the machinery can cost 5 to 10 times the yearly saving in labor. Of course, there are running and service costs on top of this, and after 10 years, a machine may need to be replaced. Where labor replacement does make sense, it is normally for factors other than direct cost, such as:

- *Labor availability* (https://www.fooddive.com/news/meat- processors-expedite-automation-as-pandemic-increases/588166/). Meat processors have spoken for many years of the difficulty in sourcing labor. For some, this has become so difficult that they are turning to automation purely to supplement recruitment efforts.
- *Health & safety*. Many tasks are hazardous to do manually, and while some Processors are focusing on the financial cost of injuries (which itself can be very high), others take more of a moral stand, deciding to reduce the risk at any cost. Full automation—removing people from the task completely—can be a solution. Other less-automated machinery can also sometimes achieve Health and Safety goals without staff removal; for example, the Bladestop bandsaw (https://www.growag.com/highlights/article/mla-funded-bladestop-a-safety-success-story; Figure 3) *that stops within milliseconds when the operator is at risk, and the Beef Boning Unit* (Figure 4) that eliminates the heavy pulling of aitchbone and knuckle pulling tasks. With these improvements in technology and safety, we can reduce absenteeism and reduce the risk for acute or chronic injuries.
- *Business continuity*. Any business must deal with fluctuations in workforce capacity. There are holiday periods, illness, staff turnover, and many other reasons for this. With meat processing being traditionally a linear process, a small number of absences can result in a much larger reduction in capacity. At no time in recent history has this been more apparent than during the Covid-19 pandemic, and, rightly or wrongly, we are seeing an increase in processors seeking to ensure continuity by reducing the reliance on a human workforce.

### Constant and consistent throughput

Closely related to labor justification, an automated production system is configured to process at a constant known speed. It never slows or stops to go to the bathroom, have a smoke or chat with a friend. Conversely, it never speeds up to get home quicker. This can have enormous benefits in downstream processes. The machinery sets the “heartbeat” of the room, removing stress on the downstream processes caused by varying production rates.

**Figure 5.**
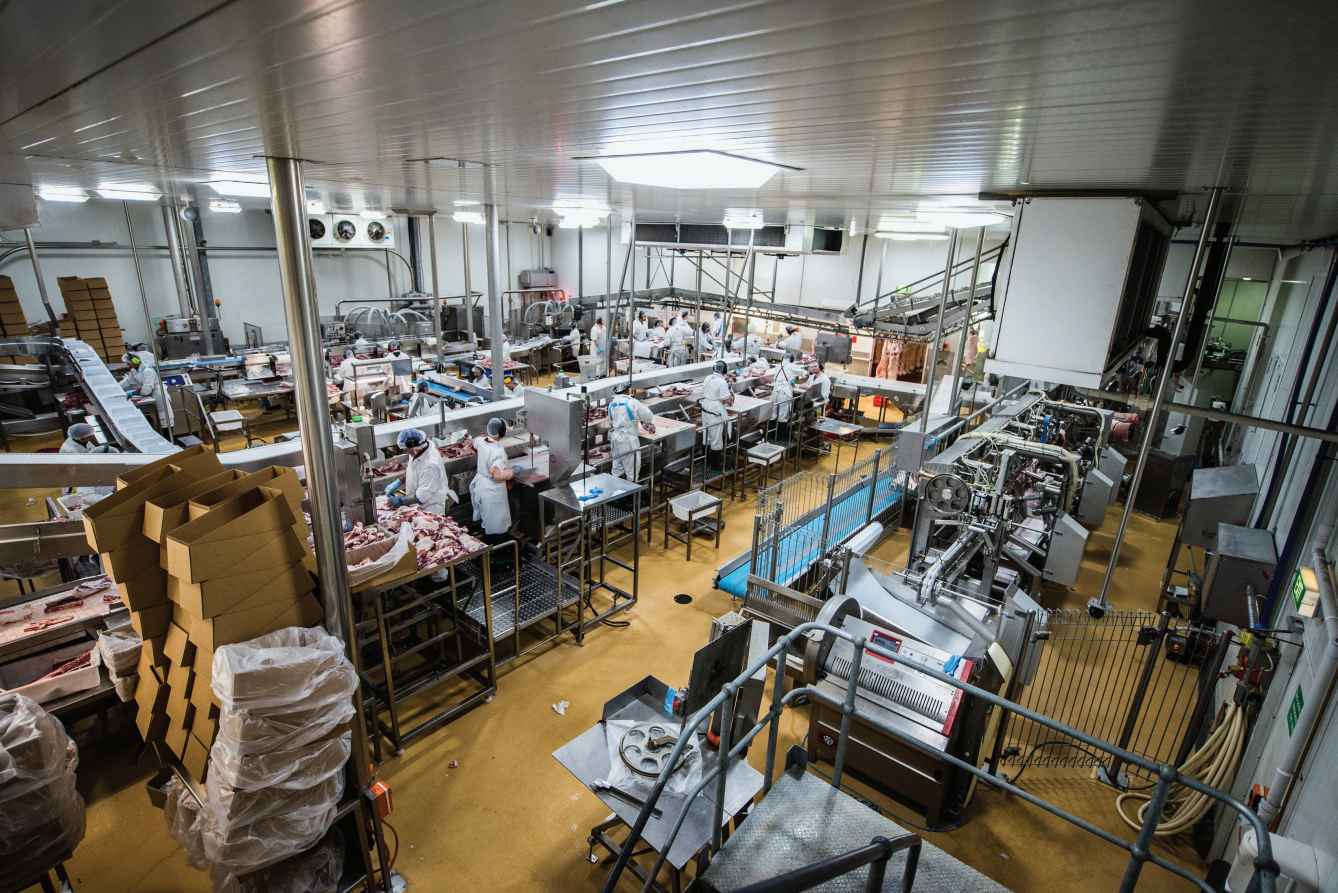
The consistent and predictable throughput of automated processing sets the “heartbeat” of the room.

We became aware of this when one of our first commercial customers for meat automation reported their experience back to us. The lamb Primal system sits in a bottleneck position in the room. Traditionally, carcasses would come to this position at a controlled, constant rate suspended from an overhead conveyor, and bandsaw operators would lift them down, cut them into three Primal sections, and then distribute these onto the correct belt conveyors for manual downstream processing. When the machine was installed, the downstream staff soon learned that a known product would arrive in a known timeframe and they were able to work in an efficient, steady rhythm. As a result, manning was adjusted to suit the set rate, rather than spikes when primal cutting was rushed. There were no pileups forcing product to drop to the floor and no rushing to cause mistakes or injuries (Figure 5).

### Product benefits

Product improvement is the most effective financial justification for automation (https://www.mla.com.au/contentassets/170c1cdc955b4f4a96202569a4ae9fff/p.pip.0327_general_leap_iii_ex_post_cba_report.pdf). If automation of a task does not result in an improved product, then there is a good chance that payback will take longer than the 2 yr payback the industry normally targets. However, some recognize that direct financial benefits are only part of the value. Key product benefits to look for are:

- *Product quality*. With automation—particularly of cutting—you are likely to see improvements in the “quality” of the product. This may be as simple as the visual aesthetics, or it may be improved contaminant control or some other variable again.
- *Shelf life*. Factors contributing to improved shelf life include lack of human contact and less surface area on the cutting face. Both factors are commonly improved through automation. Shelf life is particularly relevant for exporters who face a long freight time to get their products to market.
- *Accuracy and yield improvement.* With improvements in automated processes—particularly in sensing technologies—accuracy of automated processing is constantly improving. With improved accuracy, downstream processing can be easier, rework can be minimized, and it may be easier to meet a customer’s expected specification. But the clincher–—particularly in cutting technology—is yield improvement. Obviously in a process where bone is being discarded, there is a $ benefit in having little or no meat left on the bone (i.e., minimizing the waste). But with the value of meat on either side of a cut line varying significantly (e.g., cuts from the middle are more valuable than cuts from the adjacent forequarter), beyond keeping the cut position within spec, there is potential to bias the cut position within the specification band to be closest to the lowest value component, hence providing more of the higher value component. Yield improvement can also come from better cutting hardware. While a saw is the most common powered cutting tool, a powered knife with high cutting force provided by an automated device can do the same cut without sawdust wastage. A bandsaw blade can send as much as 2 mm per cut to waste; rotary knife cutting on lamb primals, therefore, saves 4 mm of every carcass length—lined-up back-to-back this would be 4 km of “lamb cross-section” thrown out every year. In this example, it would equate to approximately 67 tonnes of waste material reduced.

### Optimization

Production optimization is the ultimate challenge of automation but is not easy to do well.

**Figure 6.**
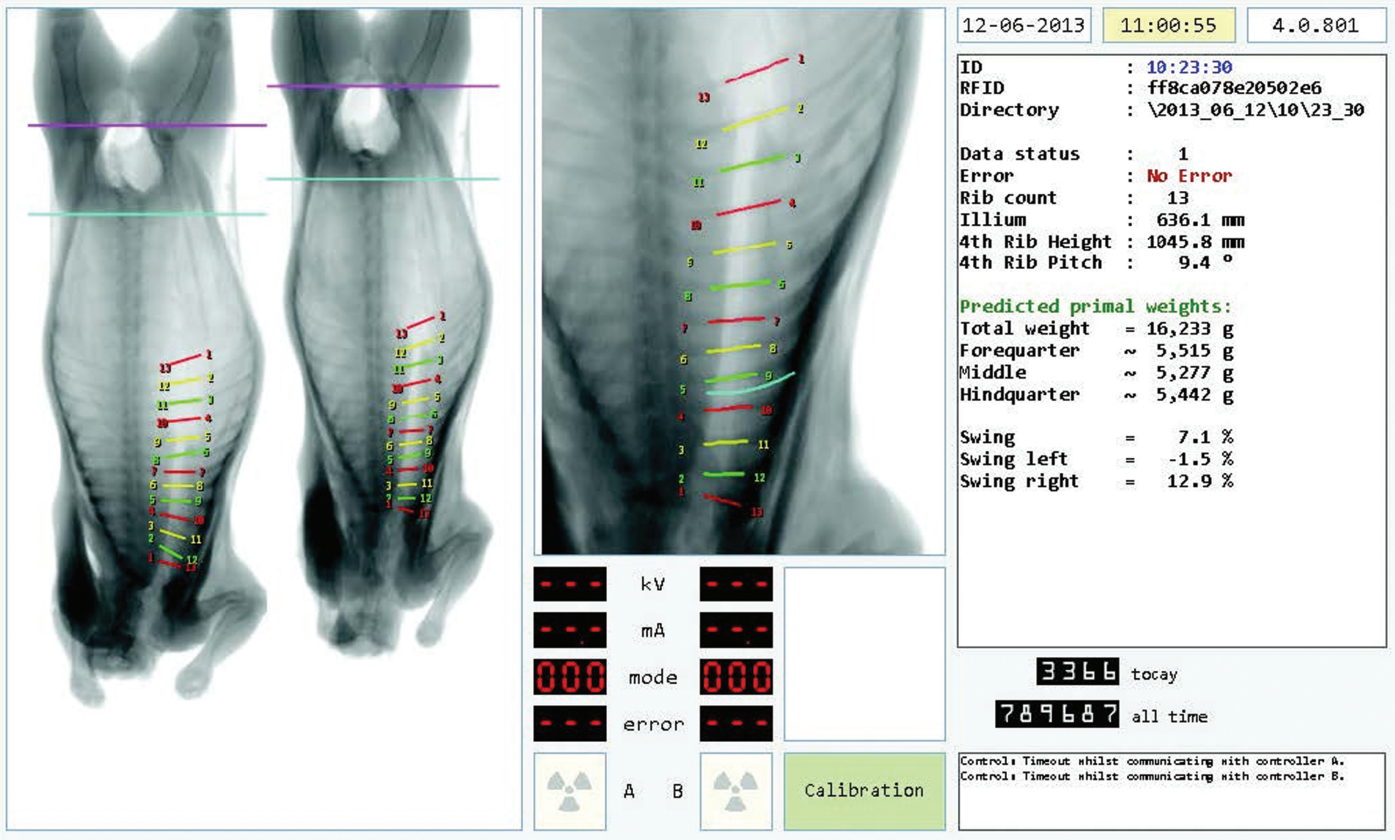
Technologies such as X-ray enable better prediction of the value of finished cuts.

Traditional grading provides the processor with a prediction of the value of a carcass to them based on the expected yield of finished cuts. With advances being seen in automated grading technologies (e.g., X-ray based as shown in Figure 6), we are getting better at predicting the expected value of the finished cuts for a range of cutting scenarios. Outwardly, processors use this as a measure for farmer payment value. But it is also used within the site to inform decision-making about how to process each carcass.

In manual processing, the processor tends to group carcasses by similar grade and can then process each grade as a batch with a particular cutting specification. But automated cutting technologies enable changing the cutting specs “on the fly.” Combining advanced grading with automated cutting allows highly flexible production to get the best yield out of every carcass, provided the processes downstream of the automated cutting can handle the variable specifications being presented to them.

Example optimization scenarios are:

- *Make to order*. Most processors work to fulfill orders of product that are determined in advance and will run each order as a batch. In the example of lamb processing, say that 50% of product sold was to include Frenched Racks and the other 50% to include Chops—both cuts from the same portion of carcass. Optimization determines the worth of each carcass under both processing options, and the highest value mix to meet the orders secured. When the whole day’s production run can be graded ahead of processing, cutting decisions can be made in advance. This has been done crudely with batch production previously, but advanced scanning and automation allow a much greater accuracy.
- *Make to sell*. We see this behavior predominantly in supermarket processors and other situations where the processor has the opportunity to influence what is offered for sale. In “Make to Order,” the processor is constrained by predetermined order quantities, whereas in “Make to Sell,” the processor will inform the market of what is available to purchase. In the scenario above, it might be determined, for example, that the best value can be achieved by processing 80% as Frenched Racks, and they will stock the shelves with this product. Of course, this only works well if the end customer always buys what is being offered…

## Adoption Considerations

Alongside the business case, there are practical considerations of planning for and adopting the automation. A useful list is below:

### Machinery size

Typically, the size of machinery necessary to automatically perform a task is greater than the amount of area used by a person performing the task manually. This can be planned for in a “Green Fields” (new build) site but may be more difficult to retrofit in an existing site.

### Throughput

With justifications being partly or wholly multiplied by the number of carcasses processed, the throughput of the line being automated should be high to ensure a short payback. Conversely, if the line throughput is greater than the capacity of the machine, it may be necessary to make a step change—e.g., adding a second machine that can double the capacity when you may only need 20% more—or look at alternative strategies such as a lower throughput manual bypass or adopting a longer shift to process the same quantity of carcasses.

### Change management

Using automated machinery in a traditionally manual processing line can be a significant change for the way a processing site is operated. This relates to all operational areas including maintenance, processing staffing, and leadership. The processor will only get the best out of automation if planning is undertaken in advance of how to restructure its operation to suit the automation.

### Breakdowns

While engineers don’t like to admit it, machines sometimes break down! This can be minimized by good design, good manufacture, and good preventative maintenance, but ultimately breakdowns can occur. In a meat processing line that can mean stopping the entire line leaving the processor with increasing costs the longer it is down. Ideally, the processor would have contingencies prepared such as the ability to bypass the machine and continue processing manually.

### Maintenance skill

When an automated processing system is installed into a processing plant, it is often the first piece of automation the processor has invested in. From a workload consisting of conveyors, bandsaws, and the like, suddenly they have a highly sophisticated robot on their hands. It is critical that this is addressed up-front—ensuring that staff are upskilled or bringing in new staff as necessary. The maintenance engineers will not be expected to delve into the software issues, but, for example, it is necessary to be able to carry out any fault-finding in a very methodical fashion. “Internet of Things” technology will help, but this cannot fully compensate for the right skills. The preference is for Service Level Agreements that include remote support, which helps to compensate for a skill mismatch at the site along with providing a means to identify any skills gaps and assist in ongoing training.

### Worker insecurity

As outlined, automation may be adopted for many reasons. But it is only natural for some staff to be concerned for their own jobs when they see automation taking on formerly manual tasks. The reality is usually different in the meat processing industry given it is often difficult to maintain sufficient manning; staff can often be redeployed to achieve efficient production volumes. The Hindquarter machine example showed a positive outcome, as a result of the workers’ insecurity. However, the processor should be prepared to manage a potentially negative response. Open communication with staff about why the automation is being adopted and what the new opportunities are for staff to work with the automation is normally the best approach.

### Product and site variability

Traditional consumer product manufacture (e.g., metal products) can rely on the consistency of the raw materials supplied to the machine. This is not so for meat processing. Mother nature is not so kind to the automation engineer, and we need to assume every single carcass is unique. And, what works for one site may not work as well for the next due to different genetics predominant at each site. Advanced sensing can overcome most of this, but in some instances, a level of algorithm retraining may be necessary, which may take time.

### Upstream processes

The output is never going to be good unless the input is good. When there is an issue showing up in the finished product or machine performance, one of the first places to look is at the carcasses coming into the system. Upstream changes aren’t always obvious in a manual line where humans will automatically compensate, but these changes can be very detrimental in an automated system. Good upstream management is important.

### Specification inflexibility

The cutting specification does not change much, but an automated system can’t necessarily just be retrained like a manual worker can. A machine may have limited flexibility based on the original specification—for example, Scott’s Primal Forequarter removal station is configured only for a planar cut which may tilt from front to back of the carcass but has no ability to roll from side to side. Its range of adjustment is only in cut height and tilt angle. This is not generally something to be concerned about, but one must be aware of the specification range the automation can cover.

## Development Considerations

For automation experts, it is largely taken for granted that what works in one industry will likely work in another. Meat processing, however, has several unique challenges to overcome. Here are our experiences:

### Location of use

The environment plays a very large part in product development, and it is important to have easy access to a typical end user location for verification. Furthermore, testing of a process requires a lot of product, and unless this is done in an approved processing plant this product is ruled unfit for consumption. We have been very fortunate in our relationships with customers who have made their plants and expertise available to us in developing solutions for them. The best scenario for us was in developing lamb processing equipment in a specially built off-production room at a Silver Fern Farms plant located only an hour from our manufacturing site. In this location, we were able to make changes behind closed doors, clean the room to hygiene standards, and then open the doors to full rate production.

### Hygiene

Designing automated equipment for a hygienic environment is extremely difficult. There are limitations in the types of materials that can be used—typically stainless steel and specific grades of plastic. Oil should be limited, and when used, it needs to be food-grade. And if material alone is not enough of a challenge, the designer needs to be looking very carefully at the design of engineering forms to ensure they are conducive to the cleaning process. 

### Cleaning implications

As well as the considerations for hygiene, the machine designer must be aware of the way in which machines are cleaned. We must allow for high-pressure water at high temperature (80 °C) as well as the colder temperatures of processing and harsh chemicals—either strongly acidic or strongly alkaline. The outcome in the field—if not properly allowed for—can be motors and other electrical/electronic devices being destroyed, rubber seals dissolved, and areas where water can pool (e.g., welds) causing rusting. Until very recently, putting an industrial robot into this environment was only possible when the robot was enclosed in a protective “suit” with positive pressure via dry air inflation to keep moisture out. Now some robots are at least being manufactured in stainless steel and seals of moving joints seem to be more suitable for our environment.

### Product variability

It is suffice to say that every product and carcass is different. What works for one carcass may not work for the next, and in development, we need to test over many products before having a reasonable level of confidence. This requires testing on-site as noted earlier and be aware that going to another plant—and worse to another country—there is a need to verify system performance at that site and potentially to make adjustments. And then once verified and running, be prepared for variability across a season. Due to this factor, it can take quite a lot longer to develop a reliable automated system for meat processing than it does for automation of homogenous product processing.

### Human vs. calculated decision-making

What we notice when we look to replicate human decision-making (e.g., cut positions) is that while one person can be better than another, overall, mistakes are made. A machine-calculated approach to decision-making will not make mistakes *based on the data it is given and the way it is trained to interpret that data.* So, a good result is seen, provided there are good data and good training. However, what this may lack is the ability to cope with anomalies in the same way that a person can—for example, a carcass with a limb missing—and some pre-sorting ability may be worthwhile. Getting the approach right means careful planning of data gathering (e.g., sensing) technologies and quite a lot of work in learning how to make the correct decisions from these data.

### Speed vs. technology

While physical machines can readily be produced to work at speed, there are always limitations. But this is a much bigger issue in sensing technologies. For obvious reasons, most of the sensing technologies we use (e.g., X-ray) have evolved from medical applications. In a medical application, there is ability to function more slowly than in a throughput-driven meat processing. And medical X-rays, for example, do not usually need to be accurate; they only give a good visual depiction of the subject. Adapting this technology is a balance between retaining the accuracy needed to guide a processing machine at throughput speed while keeping the price to an acceptable level.

### Stability

A carcass does not come in an easy shape to handle and, with inherent flexibility, can be very difficult to handle. Management of carcass stability is essential to ensure consistency of processing and full realization of automation benefits. 

## Conclusion

It is fair to say that the challenges for machine builders in developing automation and for processors in adopting it are high. But they can be overcome, and the solutions are improving all the time. With care, and the right circumstances, the justification of automation can be very compelling. Will we see full automation lights-out operations in the future? Who knows…?

## About the Author

With a mechanical engineering/design background and an emphasis on business strategy, my key areas of focus are on business and product development and customer engagement. I love working closely with customers and helping to create benefits for them. I am passionate about innovation, and I am conversant in intellectual property. At Scott Technology, we have achieved outstanding results in the innovation space, and I am very proud of my part in this; my degree is in mechanical engineering and I have also completed a Post Graduate Diploma in Executive Management, with modules in general management, leadership, strategy, and corporate entrepreneurship. Career highlights include being one of the IP creators and development initiators for Scott Technology’s X-ray, Primal system, Beef Boning Unit, and Milking Robot. I have also been closely involved in many other technology developments, and I get a lot of satisfaction in seeing the benefit these create for our customers.
